# Study on Microwave Deicing Efficiency of Microwave-Absorbing Concrete Pavements and Its Influencing Factors

**DOI:** 10.3390/ma15248923

**Published:** 2022-12-13

**Authors:** Mingyan Liu, Xuancang Wang, Yuyuan Deng, Yuchen Guo, Jing Zhao, Meixin Li

**Affiliations:** 1 School of Highway, Chang’an University, Xi’an 710064, China; 2 Shandong Provincial Communications Planning and Design Institute Group Co., Ltd., Jinan 250000, China

**Keywords:** cement concrete, magnetite, microwave deicing, microwave deicing efficiency, microwave-absorbing materials

## Abstract

Microwave deicing technology, as a new environmentally friendly deicing technology, can effectively solve the problem of the frequent icing of road surfaces in the winter, which affects the safety of traffic. To improve the efficiency of microwave deicing on cement concrete pavement, this study proposed the use of magnetite, iron sulfide slag, steel slag, lead–zinc slag, and graphite as microwave-absorbing materials, and conducted microwave deicing tests under the influence of five factors, namely the form of the pavement surface structure, the content of the microwave-absorbing material, microwave power, the shielding state, and dry and wet conditions. Layer by layer, we selected the combination of pavement surface structure, microwave-absorbing material content, microwave power, shielding state, and dry and wet conditions on the bottom surface of the concrete slab with the optimal deicing effect. The results showed that the 2 cm scattered microwave-absorbing surface concrete structure has the fastest heating rate; the higher the magnetite content and microwave power, the higher the deicing efficiency; the maximum heating rate can be increased by 17.6% when the shielding layer is set at the bottom of the cement concrete slab; and the heating rate of the microwave-absorbing concrete slab in the wet state is increased by 20.8% relative to the dry state. In summary, 7000 W of power, a magnetite content of 60 vol % in the scattered microwave-absorbing surface, a shielding layer set at the bottom surface, and wet conditions can greatly improve the efficiency of microwave deicing compared with the microwave ice melting effects of plain cement concrete and other microwave-absorbing materials mixed into the concrete. In addition, the temperature uniformity of the microwave-absorbing materials is essential to improve the deicing efficiency of microwave-absorbing concrete, so it is essential to explore it further.

## 1. Introduction

Snow and ice on roads in the winter can greatly reduce the friction coefficient of the road’s surface. On icy roads, vehicles may have accidents due to difficulties in braking [1], and aircraft may skid during takeoff and landing, reducing their flight speed and even causing accidents [2,3]. To ensure the safe operation of important transportation facilities such as highways and airports, it is especially important to remove ice from the road surface quickly and effectively. The current deicing methods used for pavement are mainly cold air deicing, chemical deicing, manual deicing, mechanical removal, etc. [4,5]. However, these methods are harmful to the pavement structure and the ecological environment to some extent. The cold air deicing method may damage the pavement surface at low temperatures, chemical deicing using salt will break the structure of the concrete and pollute the environment, and manual deicing methods or mechanical deicing methods are costly in terms of labor and material resources and have low efficiency [6,7,8].

Microwave deicing, as a new environmentally friendly deicing technology, has the advantages of a high removal rate and no damage to the pavement surface, and it has good prospects for development [9]. The principle is to use microwaves to increase the temperature of the pavement surface, so that the ice melts and is stripped from the pavement surface, and then the ice is cleared with the help of other machinery to ensure the safety of traffic. This technology has received wide attention from relevant experts and scholars [10]. In the 1980s, the theory of using microwave heating combined with mechanical deicing devices to remove accumulated road ice was proposed in the United States, and related research and experiments were conducted [11]. However, this method is not widely used because of its low deicing efficiency. In the 21st century, scholars from various countries have shifted their research focus to enhancing the efficiency of microwave deicing by incorporating microwave-absorbing materials. Guan et al. [12] experimentally proved that a mixture of microwave-absorbing materials can enhance the ice melting effect on the road’s surface, and designed a schematic diagram of a microwave deicing vehicle. Zanko et al. [13] applied iron–copper ore to asphalt pavements to study the on-road performance and microwave absorption properties of asphalt pavements in depth. Hopstock et al. [14] used iron flint as a microwave-absorbing material for an asphalt pavement surface layer and found that the microwave absorption capacity of the pavement’s surface layer was enhanced and the efficiency of microwave deicing was improved. Gao et al. [15,16] studied the mechanism of microwave deicing, mixed steel slag into an asphalt mixture to improve the efficiency of ice melting on the pavement, and looked forward to further developments of microwave deicing. Gallego et al. [17] added 0.2% steel fibers to asphalt concrete and concluded that steel fibers increased the sensitivity of asphalt concrete to electromagnetic induction. Rajavaram et al. [18] studied the microwave heating properties of magnetite in depth and concluded that microwave power and the magnetite’s quality had a large effect on its properties. Guan et al. [19] experimentally studied the microwave heating performance of MPAM (Asphalt Mixture Mixed with Natural Magnetite Powder) and found that its microwave absorption performance was affected by the thickness and the microwave frequency in the structural layer of the pavement. Wang et al. [20,21] conducted experiments on microwave deicing efficiency and the durability of concrete with different magnetite contents, and obtained that magnetite can improve the wear resistance and compressive strength of concrete and that the deicing effect is gradually enhanced with the increase of magnetite content. Liu et al. [22] systematically analyzed the deicing efficiency and mechanical properties of iron black concrete formed with different amounts of iron black admixtures and found that the mechanical properties of concrete decreased with an increase in the iron black admixture, but that the microwave deicing efficiency was improved to a great extent. Natt Makul et al. [23] systematically studied the applications of microwave energy in the field of cement, describing the influence of the dielectric properties of materials on the efficiency of microwave heating. Wang et al. [24] proposed a microwave snow and ice removal technology and selected two types of hydroxy iron powder (RW and RW1), tri-iron tetroxide, alumina and expanded graphite, as microwave-absorbing materials in a deicing test of microwave-sensitive coating materials. The results showed that microwave deicing was more efficient than traditional concrete deicing. Jiao et al. [25] conducted simulations and experimental studies at ambient temperatures and concluded that the efficiency of microwave deicing decreases with a decrease in the ambient temperature. Ding et al. [26] used finite element simulation software to establish a model to simulate and analyze indoor microwave deicing experimental data, and the results showed that microwave deicing efficiency was greatly influenced by microwave frequency and road structure materials. Chen et al. [27] studied the heating law of concrete surfaces and the influencing factors in the process of deicing based on the principle of microwave de-icing technology and developed a microwave deicing test system suitable for airport road surfaces. Huang et al. [28] designed experiments to study the microwave deicing law of carbon fiber-modified concrete under the effects of multiple factors, obtained that the length and amount of carbon fiber affect the microwave deicing efficiency, and deduced the relationship between microwave deicing and the rate of temperature increase. Xia et al. [29] optimized the shape and size of the horn antenna on the microwave source and designed an experiment to compare and analyze the effects of the width and height of the horn antenna on the efficiency of microwave ice melting. The above research achievements to a large extent promote the development of microwave deicing technology for roads. However, there are still some gaps in the above research, mainly the following: First, most scholars have studied the microwave deicing of asphalt concrete pavements. However, the properties of asphalt concrete and cement concrete are very different, so the microwave deicing law of asphalt concrete does not apply to cement concrete, and it is necessary to do further research on the microwave deicing of cement concrete. Second, the above research has mainly revolved around microwave-absorbing materials, the efficiency of melting ice, and other directions. However, there is a lack of research that comprehensively considers the structural form, material, microwave power, and road conditions during microwave deicing under the action of multiple influencing factors. Therefore, there is great value in studying the microwave de-icing efficiency of microwave-absorbing concrete pavements under the effects of multiple factors.

In order to solve the above-mentioned problems, this study selected five microwave-absorbing materials for research, considering the influence of the form of the pavement’s surface structure, the content of the microwave-absorbing materials, microwave power, the shielding state, and dry and wet conditions on the microwave deicing efficiency of a microwave-absorbing concrete road surface. We optimized these factors layer by layer and finally determined the combination of microwave-absorbing concrete with the optimal deicing effect. The results of this study can provide a reference for the microwave deicing of ice-prone concrete pavements in cold areas, greatly improving the efficiency of microwave deicing and the timely removal of road ice to ensure traffic safety.

## 2. Materials and Methods

### 2.1. Raw Materials

In this paper, ordinary PC 42.5 silicate cement was produced by Wuxi Jianghuai Building Materials Technology Co., Wuxi, China, and its basic properties are shown in Table 1. The coarse aggregate was common limestone gravel aggregate, and its basic properties are shown in Table 2. The fine aggregate was river sand, and its basic properties are shown in Table 3. The test materials all met the requirements of the relevant specifications of the Technical Rules for the Construction of Highway Cement Concrete Pavements (JTGT F30-2014).

**Figure 1:**
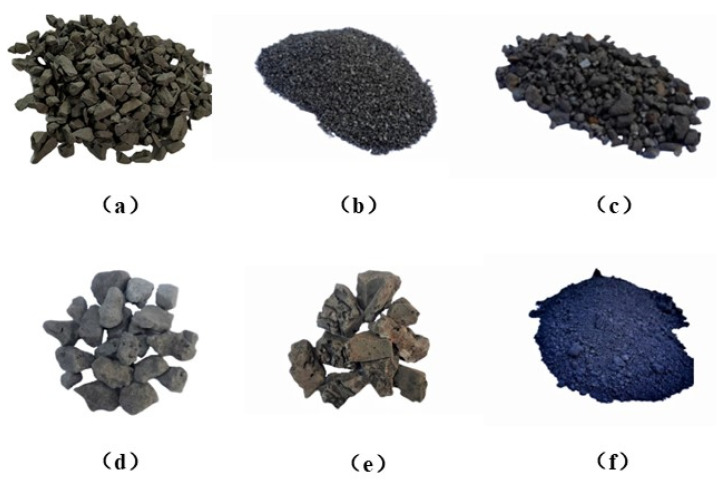
Microwave-absorbing materials used in the test: (**a**) magnetite; (**b**) 1–3 cm iron sulfide slag; (**c**) 5–10 cm iron sulfide slag; (**d**) steel slag; (**e**) lead–zinc slag; (**f**) graphite powder.

In this study, five microwave-absorbing materials, namely magnetite, iron sulfide slag, steel slag, lead–zinc slag, and graphite powder, were used for the tests. The magnetite ore was produced in Baotou, Inner Mongolia; the low-grade iron sulfide slag was produced in Tongling, China; the continuous-grade steel slag was produced by Handan Iron and Steel Plant, Handan, China; the continuous-grade lead–zinc slag was produced in Baiyin, China; and the graphite powder was produced by Huabang Mineral Products Company, Shijiazhuang, China. These materials are shown in Figure 1 and their basic properties are shown in Table 4.

### 2.2. Specimen Preparation

**Figure 2:**
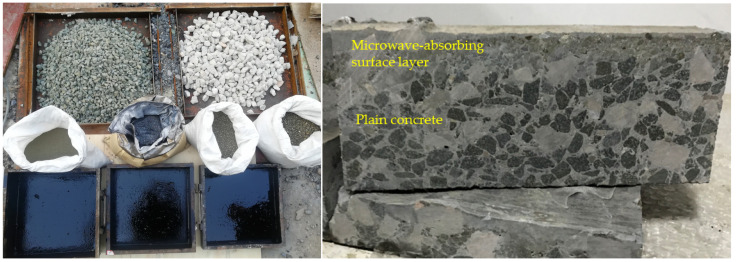
Raw materials of the scattered microwave-absorbing surface and the microwave-absorbing concrete specimens.

The microwave-absorbing materials (magnetite, iron sulfide slag, steel slag, lead–zinc slag, and graphite) were designed in line with the principle of equal volume substitution, and the limestone gravel was mixed with equal volume substitution at different substitution rates. This study proposed two types of microwave-absorbing road surface layer structures, namely, a scattered microwave-absorbing surface layer and a thin double-layered microwave-absorbing concrete. The surface of the scattered microwave-absorbing structure was a microwave-absorbing fine-particle aggregate, and a microwave-absorbing powder was scattered directly and evenly on the concrete’s surface during the process of forming the cement concrete, so that the microwave-absorbing material could be both integrated within the concrete slab and evenly distributed on the concrete’s surface, forming a functional surface layer of concrete with deicing as its core purpose, as shown in Figure 2. Each microwave-absorbing material was formed into concrete specimens with microwave-absorbing functional layers of different thicknesses; the thickness of the entire microwave-absorbing concrete road surface was 2 cm, 3 cm, or 4 cm.

The structure of the thin double-layered microwave-absorbing concrete was a thin layer and a lower bearing layer. The thin double-layer microwave-absorbing concrete was formed with two ratios, layered pounding and splitting into the mold. In other words, plain concrete and the thin microwave-absorbing concrete layer were poured twice to the specified size and pounded in layers. The specific preparation steps involved the following: (a) The lower bearing layer of ordinary concrete is loaded into the test mold. The spatula should be inserted and pounded along the inner wall of the test mold when loading the material. (b) The test mold is attached or fixed to the vibrating table, where the vibration should continue until the surface of the pulp has no obvious large bubble overflow. Do not over vibrate while waiting for the initial setting. (c) Pour the microwave-absorbing layer of concrete into the mold so that the concrete mixture is above the top opening of the test mold and vibrate it tightly again according to step (b).

**Figure 3:**
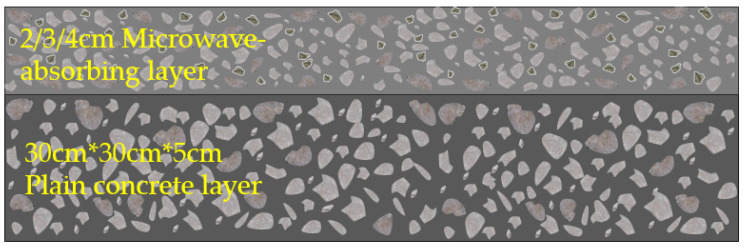
Schematic diagram of double-layer microwave-absorbing thin-layer concrete specimen.

The schematic diagram of the double-layer microwave-absorbing thin-layer concrete specimen is shown in Figure 3. The specimen was kept static for two days and nights, and then, after it had been removed from the mold, it was placed in a maintenance room with a standard constant temperature and humidity for 28 d (Temperature: 20 °C ± 2 °C; Humidity: above 95 % RH). The specimen was frozen in the freezer at a temperature of −10 °C for 48 h. The process of specimen preparation, maintenance temperature, and humidity all met the requirements of the relevant specifications of the Standard for Test Methods of Physical and Mechanical Properties of Concrete (GB/T50081-2019).

### 2.3. Test Equipment and Test Method

#### 2.3.1. Test Equipment

In this study, a QW-10HO high-power industrial microwave cabinet produced by Guangzhou Dewei Industrial Microwave Equipment Co., Guangzhou, China was selected for the microwave heating test of the microwave-absorbing concrete specimens. The microwave cabinet heating had a cavity size of 100 cm × 87 cm × 55 cm. The microwave cabinet’s cavity was fitted with a set of 1000 W rectangular waveguide ports; the working power could be adjusted to three levels, namely 4000 W, 7000 W, and 10,000 W; the microwave frequency was 2.45 GHz.

**Figure 4:**
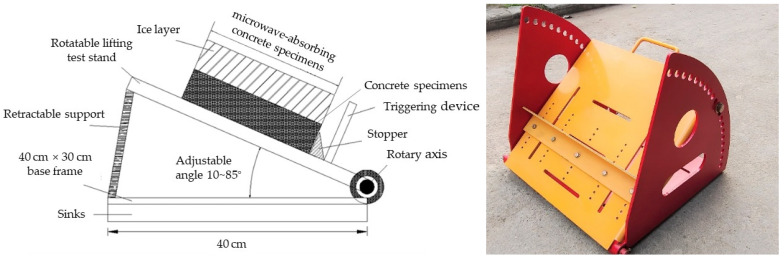
Design diagram of the new device for testing the efficiency of microwave deicing and the physical device.

Our group designed and developed a test device dedicated to measuring the efficiency of microwave deicing, as shown in Figure 4. The specific use of the device is to turn on the automatic timer while heating the microwave-absorbing concrete specimen with a certain thickness of ice layer in the microwave cabinet. The surface of the specimen of microwave-absorbing material was heated after absorption of the microwaves, causing melting at the ice–specimen interface, and the ice was gradually stripped from the surface of the microwave-absorbing concrete specimens. The ice slipped and hit the trigger of the alarm device, telling the automatic timer to stop timing. To simplify the experiment, the microwave deicing time was recorded by manual observation and timing, and the length of time between the start of the ice sliding and the complete sliding of the ice (called sliding time) was used to characterize the efficiency of microwave deicing by different microwave-absorbing concrete specimens.

In this study, the SMART SENSOR AT 390 infrared thermometer produced by SMART SENSOR, Hong Kong, China was selected for concrete slab surface temperature measurement, and its main technical parameters are measurement temperature range: −32 ~ 380 °C; measurement accuracy: ±2 °C/± 2%; resolution: 0.1 °C/0.1 °F; response time: (8–14) μm and 500 ms; repeatability: ±1% or ± 1 °C.

#### 2.3.2. Test Method

**Figure 5:**
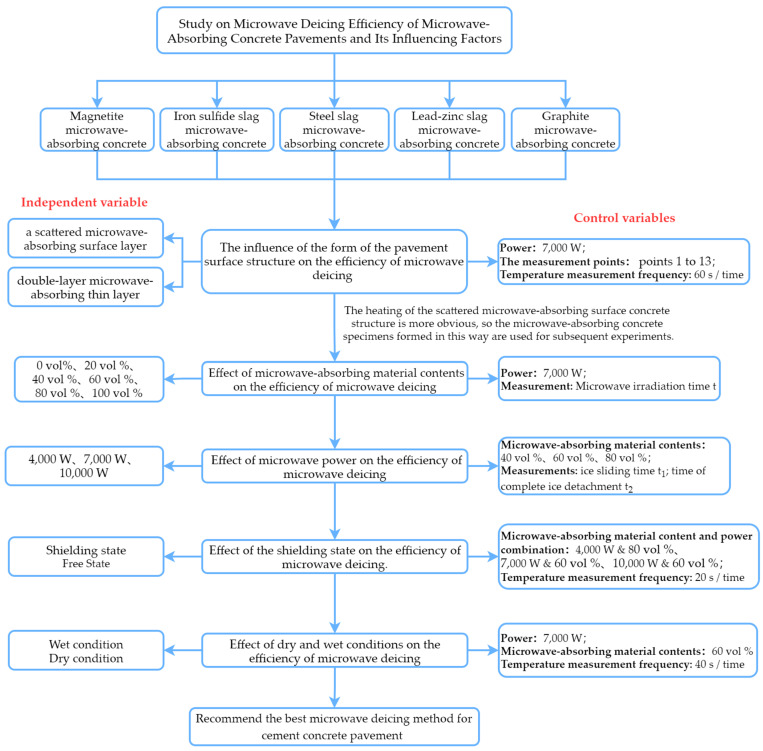
Experimental flow chart of microwave deicing efficiency and influencing factors of microwave-absorbing concrete road surface research.

This study mainly investigated the influence laws of microwave deicing efficiency; the specific experimental procedure is shown in Figure 5.

**Figure 6:**
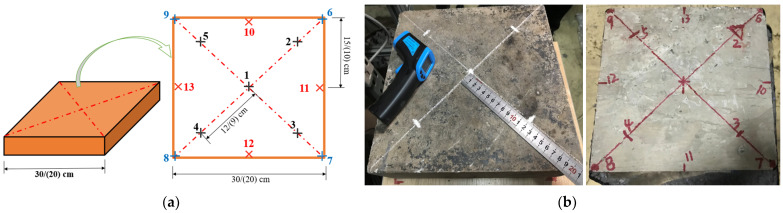
Arrangement of the measurement points on the surface of the concrete specimen: (**a**) Schematic diagram of concrete specimen; (**b**) Physical drawing of concrete specimen.

In the process of conducting tests on the effect of the structural form of road surface, dry and wet conditions, and the shielding condition on the microwave deicing efficiency, the temperature changes at different locations on the concrete slab were used as the evaluation criteria. The specific test method was as follows: the temperature was measured once every 60 s during heating, and the temperature data of different positions with the increase in the microwave heating time were recorded. The temperature measurement points were marked at different locations on the board, as shown in Figure 6, where temperature measurement Point 1 is the center of the board, Points 2–5 are the surface temperature measurement points on the board, Points 6–9 are the temperature measurement points on the corners of the board, and Points 10–13 are the temperature measurement points at the edge of the board.

**Figure 7:**
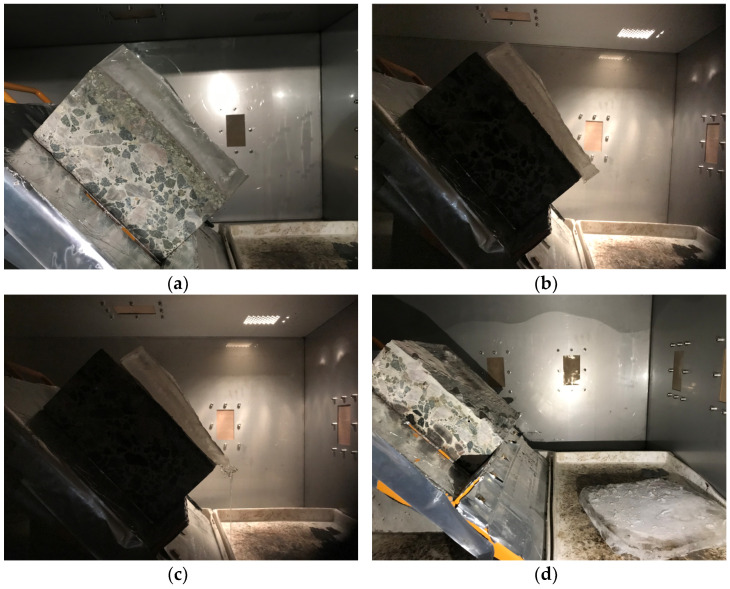
The process of the microwave deicing efficiency test: (**a**) frozen cement concrete specimen with ice; (**b**) ice in the sliding state; (**c**) ice in the process of sliding; (**d**) completely detached ice.

The efficiency of microwave deicing was defined as the speed at which the ice layer changes from a frozen state to a separated state at the point of contact between the road’s surface and the ice layer, that is, the time (t, in seconds) required for the microwaves to melt the ice layer at the road–ice contact surface. In order to test the influence of microwave-absorbing material content and microwave power on the efficiency of microwave deicing, it is necessary to freeze the 3 cm thick ice layer on the concrete slab and take the sliding time of the ice layer as the evaluation standard. The test process is shown in Figure 7.

The five tests covered in this study had the following requirements for the specimens during the test.

**Figure 8:**
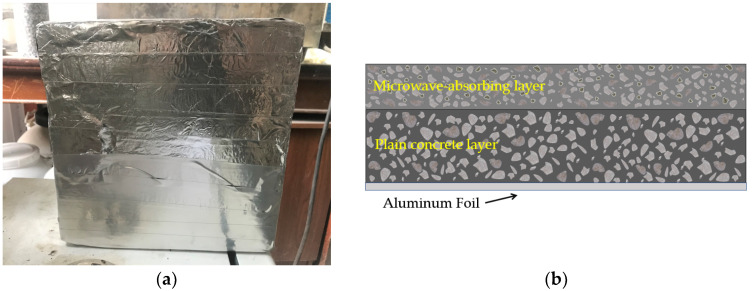
Microwave-absorbing cement concrete with shielding at the bottom surface: (**a**) Bottom surface shielded concrete specimen. (**b**) Schematic diagram of the bottom shielded concrete specimen.

(a) For the test of the form of the road’s surface structure, specimens of the scattered microwave-absorbing surface with thicknesses of 2 cm, 3 cm, and 4 cm and a magnetite content of 60 vol %, and specimens of the thin double-layered microwave-absorbing surface with thicknesses of 2 cm, 3 cm, and 4 cm and a magnetite content of 60 vol % were formed according to the methods proposed in Section 2.2, followed by the microwave heating experiments. The temperatures at different locations on the concrete slabs’ surfaces, i.e., at Points 1 to 13, were recorded throughout the test, and the average value of the temperature at all points was taken.
(b) In the tests of the content of the microwave-absorbing material, magnetite was used to replace crushed limestone aggregates at 0 %, 20 %, 40 %, 60 %, 80 %, or 100 % by volume. Microwave-absorbing concrete slabs (2 cm in thickness) were made according to the method of forming the scattered microwave-absorbing surface specimens.
(c) In the test to investigate the effect of microwave power, the microwave power was set to 4000 W, 7000 W, and 10,000 W. Microwave heating experiments were carried out on cement concrete specimens with a scattered microwave-absorbing layer with different magnetite contents (40 vol %, 60 vol %, and 80 vol %) at different levels of microwave power.
(d) In the test of the shielding conditions, according to the dissipation and loss path of microwaves in the cement surface of the road, some of the microwaves will penetrate the microwave-absorbing cement concrete slab and reach the next structural layer of the concrete slab. Therefore, this study used metal aluminum foil as a microwave shielding material pasted underneath the cement concrete slab to simulate the state of the cement concrete slab at the bottom shielding layer, as shown in Figure 8, to further examine the efficiency of the heating by microwave absorption in a microwave-absorbing cement concrete slab with bottom shielding. Before the test, the concrete specimens with different magnetite contents were divided into those in the fully free state and those with bottom shielding, and the average value of the temperature measured at five points on the concrete slab (Points 1–5) was taken as the object of analysis and comparison in order to study the influence law of the surface heating rate of microwave-absorbing concrete in the two states (free and with bottom shielding).

(e) The four tests described above were carried out in a dry environment, but the actual process of microwave deicing in a microwave-absorbing concrete road surface can generate its own heat through the action of the microwave-sensitive materials. After the heat has been transferred to the road–ice interface, the frozen ice at the point of contact between the road’s surface and the ice layer will gradually melt, producing water, and the microwave-absorbing road surface will become wet. Therefore, in this test, the effect of the moisture in the microwave-absorbing concrete on the efficiency of microwave deicing was analyzed by comparing the data on the increase in the microwave absorption temperature for dry and wet concrete slabs. The microwave-absorbing material contents were 60 vol % magnetite, iron sulfide slag, steel slag, lead–zinc slag, or graphite made into specimens with a scattered microwave-absorbing layer. These specimens were placed in a water bath for 6 h, and the surface moisture of the specimen was wiped dry. The concrete specimens were heated by a 7000 W microwave in both dry and wet conditions, and the average values of the temperatures measured at five points (Points 1–5) on the concrete slab in order to study the absorption rate of the microwave-absorbing concrete in dry and wet conditions.

## 3. Results

In this study, five microwave-absorbing materials were used to form concrete specimens for testing the effects of the form of the road’s surface structure, the content of the microwave-absorbing material, microwave power, and the shielding condition on the efficiency of microwave deicing. The results show that the law of deicing efficiency of the five microwave-absorbing materials was similar for four factors, and that the laws for steel slag and magnetite changed most significantly. However, during the microwave deicing test, the warming of the steel slag was a non-uniform; specifically, the center point’s temperature was higher, the rate of warming was fast, and temperature stress was easy to produce in the pavement, which could cause cracking. Therefore, this study considered the changes in the microwave deicing performance of magnetite microwave-absorbing concrete under the influence of different factors as an example to analyze the trends of the different factors in the microwave deicing performance. Considering the environmental factors in an actual road, a test of the effect of dry and wet conditions on the efficiency of microwave deicing was carried out for all microwave-absorbing materials. In the tests, data on the time when the ice started sliding, the complete sliding time, and the different temperatures on the specimens’ surfaces during the microwave deicing process were measured. The data were processed and analyzed to compare the change trends, and the following results were obtained.

### 3.1. The Influence of the Form of the Pavement Surface Structure on the Efficiency of Microwave Deicing

**Figure 9:**
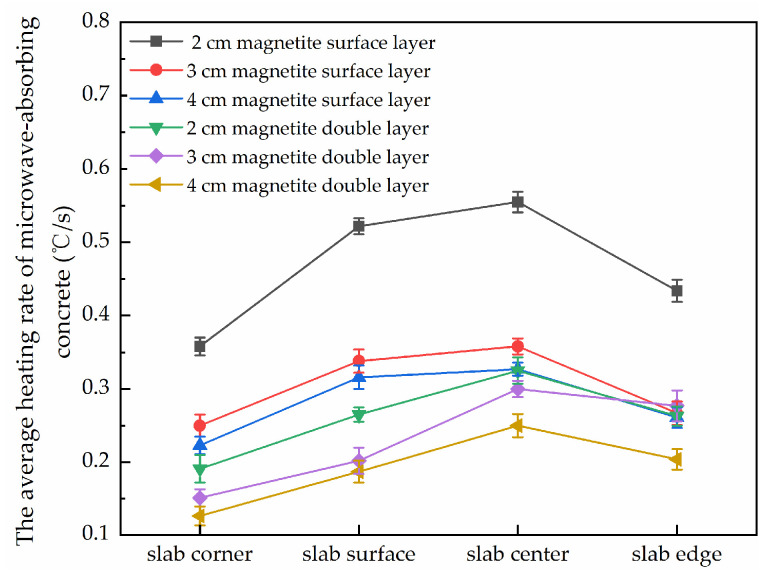
The average heating rates at different positions for concrete slabs with different microwave-absorbing materials (°C/s).

After processing the data measured during the test, the heating rate at different locations of the concrete slab was obtained, as shown in Figure 9.

As can be seen in Figure 9, under the same microwave heating conditions, at the center of the slab and at the other locations on the slabs’ surfaces, the better warming rate of the scattered microwave-absorbing surface structure was obvious. The warming rates of all three thicknesses of scattered microwave-absorbing surface concrete were higher than those of the double-layer microwave-absorbing concrete structure. The main reason for this is that the microwave-absorbing magnetite material was uniformly distributed on the surface of the test piece and could directly receive the microwave radiation [14,19], and thus the specimen could heat without heat conduction and the utilization rate of the microwave energy was high. On the other hand, the microwaves were reflected less by the surface of the thin double-layered microwave-absorbing specimen, and more microwaves entered the microwave-absorbing concrete specimen, which increased the microwave utilization rate, but the main body of this structure was still made up of microwave-absorbing materials, and thus required continuous heat transfer to the surface of the concrete specimen by the microwave-absorbing materials, and so the energy utilization rate was relatively low compared with the scattered microwave-absorbing specimen, and the heating rate of the microwave-absorbing surface was also relatively slow.

Decreasing the thickness of the magnetite can significantly improve the microwave heating capacity of asphalt mixes [30]. From Figure 9, it can be seen that the 2 cm specimens with magnetite and a scattered microwave-absorbing surface structure had more significant warming rates at different locations. The 2 cm magnetite surface note only absorbed the microwaves the surface of the test specimen but simultaneously received the incident microwaves from the bottom of the plate inside the microwave cabinet’s resonant cavity during heating. Due to its thin thickness, when the internal loss of incident microwaves from the bottom of the test piece is small, more microwaves will reach the upper surface of the test piece and be absorbed by the magnetite, achieving a higher microwave utilization rate. Therefore, all subsequent experiments in this study were conducted using a scattered microwave-absorbing surface concrete structure.

### 3.2. Effect of Microwave-Absorbing Material Contents on the Efficiency of Microwave Deicing

**Figure 10:**
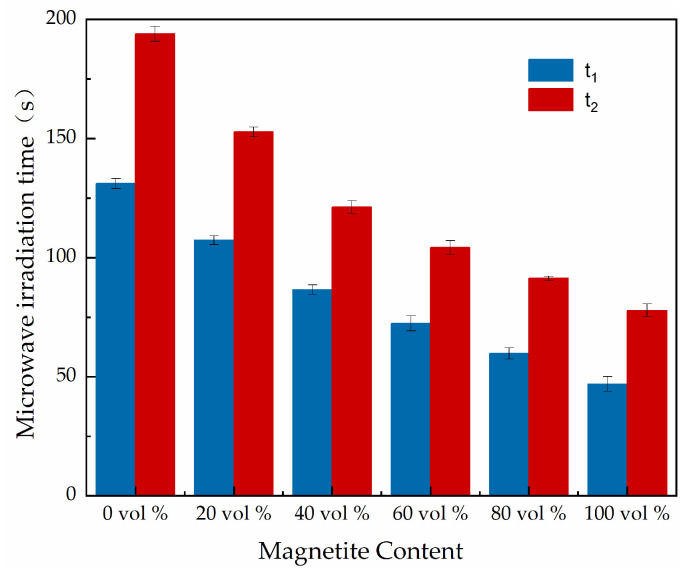
Experimental results of the deicing efficiency of microwave-absorbing concrete specimens with different magnetite contents.

Microwave deicing experiments were conducted on concrete slab specimens with different magnetite contents, and five parallel groups of specimens were prepared for each content. Here, t_1_ and t_2_ were defined as the time when the ice layer started to slide and when it completely detached from the concrete specimen. The concrete needs to be vibrated during the forming process, and the upper surface layer is mainly dominated by cement paste. Therefore, the friction generated on the contact surface between the concrete surface of different microwave-absorbing materials and the ice layer is approximately the same, and t_1_ and t_2_ can be used to characterize the microwave deicing efficiency of the microwave-absorbing concrete. The test results are shown in Figure 10.

Wang et al. prepared specimens of 0%, 20%, 40%, 60%, 80%, and 100% content magnetite concrete; Liu et al. prepared iron black concrete with different content of iron black molding; and Meng et al. prepared carbon fiber modified concrete specimens with 1%, 2%, and 3% concrete volume to study the effects of different contents of magnetite, iron black, and carbon fiber on the microwave deicing efficiency of concrete, and the results showed that the increase of magnetite, iron black, and carbon fiber content can substantially improve the microwave ice melting efficiency of cement concrete [20,21,22,31]. In Figure 10, it can be seen that the deicing time (t_1_ and t_2_) of the microwave-absorbing concrete specimens showed a significant decreasing trend with an increase in the magnetite admixture in the concrete. The microwave deicing efficiency was improved. The time to start sliding and the time to complete detachment were reduced by 16.4% and 31%, and 14% and 24.7%, respectively, for 60 vol % and 80 vol % admixtures of magnetite concrete relative to the 40 vol % admixture of magnetite, and were reduced by 32.6% and 44.3%, and 31.8% and 40.3%, respectively, relative to the 20 vol % admixture of magnetite. The main reason for this is that the admixture of magnetite can significantly improve the dielectric loss of microwaves of the cement concrete; can, to a certain extent, improve the magnetic polarization loss of microwaves in the concrete; and can reduce the gap between dielectric loss and magnetic polarization loss on the surface of the cement concrete, which is conducive to improving the impedance matching characteristics of the concrete surface and reducing the reflectivity of microwaves on the concrete’s surface, thus enhancing the microwave absorption and warming efficiency of the cement concrete [32,33,34]. The test results also showed that the microwave deicing rate was faster when the magnetite content increased from 40 vol % to 60 vol % compared with the increases in magnetite content from 20 vol % to 40 vol % and from 60 vol % to 80 vol %. Therefore, this study conducted tests on the effect of microwave power on microwave deicing efficiency for concrete specimens with magnetite contents of 40 vol %, 60 vol %, and 80 vol %, with the aim of selecting a suitable magnetite content.

### 3.3. Effect of Microwave Power on the Efficiency of Microwave Deicing

**Figure 11:**
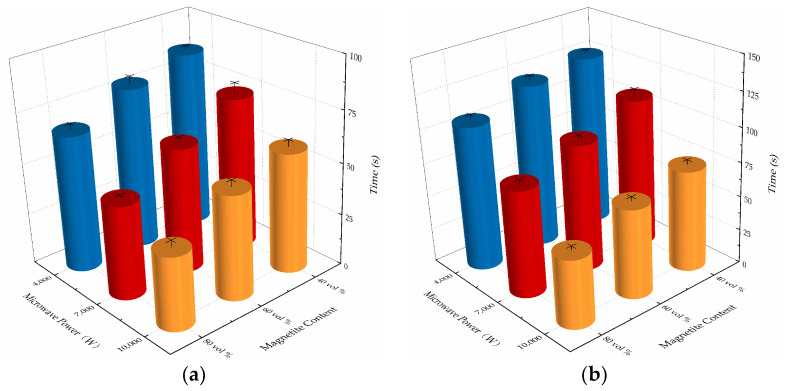
Experimental results of the deicing efficiency of concrete with different microwave-absorbing materials content and different levels of microwave power: (**a**) sliding time t_1_ (s); (**b**) time of complete ice detachment t_2_ (s).

**Figure 12:**
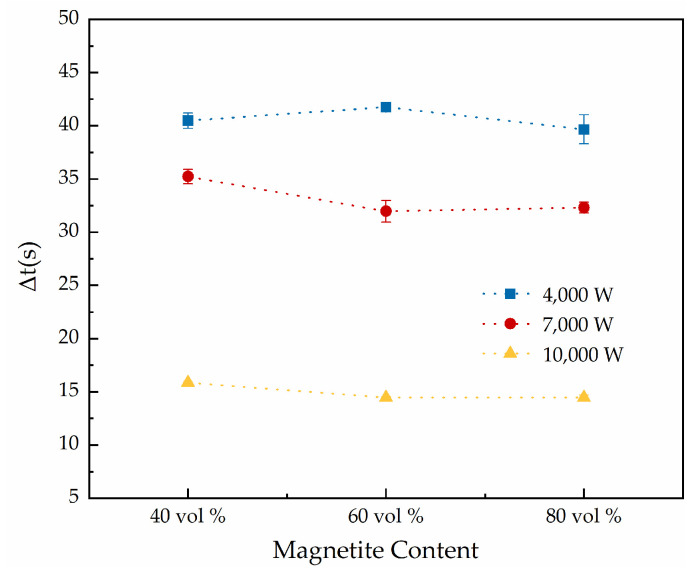
Trend of Δt under different levels of microwave power and different magnetite contents.

Here, Δt is the difference between t_2_ and t_1_, which is the time from the start of the ice sliding to complete removal of the ice from the microwave-absorbing concrete specimens. The data on the deicing time (t_1_, t_2_ and Δt, in s) for the cement concrete specimens with different magnetite content (40 vol %, 60 vol %, and 80 vol %) were as shown in Figure 11 and Figure 12. (Figure 10: x-axis: magnetite content; y-axis: microwave power; z-axis: mean value of time).

Rajavaram et al. studied the microwave heating characteristics of magnetite concrete at 1500, 1600, and 1700 W power; Ye et al. applied finite element software to simulate the microwave heating effect of magnetite at 800, 1000, 1200, and 1500 W; and Luo et al. designed a microwave deicing device based on the principle of the microwave generator set and studied the effect of microwave power on the deicing effect. The results showed that, with the increase of microwave power, the microwave deicing efficiency was significantly improved [18,35,36]. The inverse of the ice sliding time t_1_ was defined as the efficiency of microwave deicing. As can be seen in Figure 11, at a microwave power of 4000 W, the deicing efficiencies of the 60 vol % and 80 vol % magnetite were 1.197 times and 1.449 times that of the 40 vol % magnetite. At a microwave power of 7000 W, the deicing efficiencies of the 60 vol % and 80 vol % magnetite were 1.250 times and 1.602 times that of the 40 vol % magnetite. At a microwave power of 10,000 W, the deicing efficiencies of the 60 vol % and 80 vol % magnetite were 1.250 times and 1.602 times that of the 40 vol % magnetite.

In Figure 12, it can be seen that the difference in Δt among the concrete slabs with different magnetite contents was small under the same microwave power, but that the difference in Δt was large under different levels of microwave power. With a magnetite content of 60 vol %, the average value of Δt at 7000 W decreased by 19.27 % relative to that at 4000 W, while the average value of Δt at 10,000 W decreased by 57.10 % relative that at 7000 W. All of the above data showed that, in the process of microwave deicing of cement concrete pavement, increasing the microwave power can enhance the efficiency of microwave deicing and shorten the time used to deice. The reason is that the liquid water formed from the melting ice absorbs a large number of microwaves, and thus the influence of microwave power is more obvious, and with an increase in the power of the incident microwaves, the heating rate is significantly increased and the freezing force of the road–ice interface decreases more greatly; that is, the performance of Δt was significantly reduced with an increase in the microwave power. Therefore, increasing the microwave power can improve the efficiency of microwave deicing in the process of the microwave deicing of cement concrete pavement.

According to the test results in this section, we selected 80 vol % magnetite at a power of 4000 W, 60 vol % magnetite at a power of 7000 W, and 60 vol % magnetite at a power of 10,000 W for the next test.

### 3.4. Effect of the Shielding State on the Efficiency of Microwave Deicing

**Figure 13:**
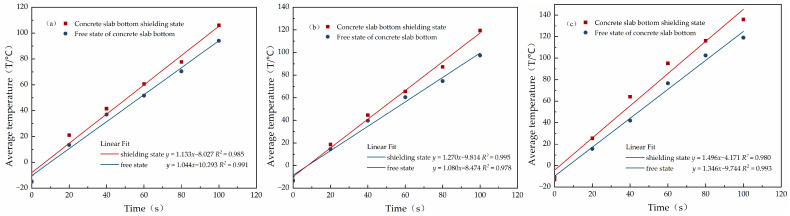
Linear fit of the mean value of the surface temperature for concrete slabs with different magnetite contents at different levels of power in the free state and with and bottom shielding: (**a**) 4000 W and 80 vol %; (**b**) 7000 W and 60 vol %; (**c**) 10,000 W and 60 vol %.

**Figure 14:**
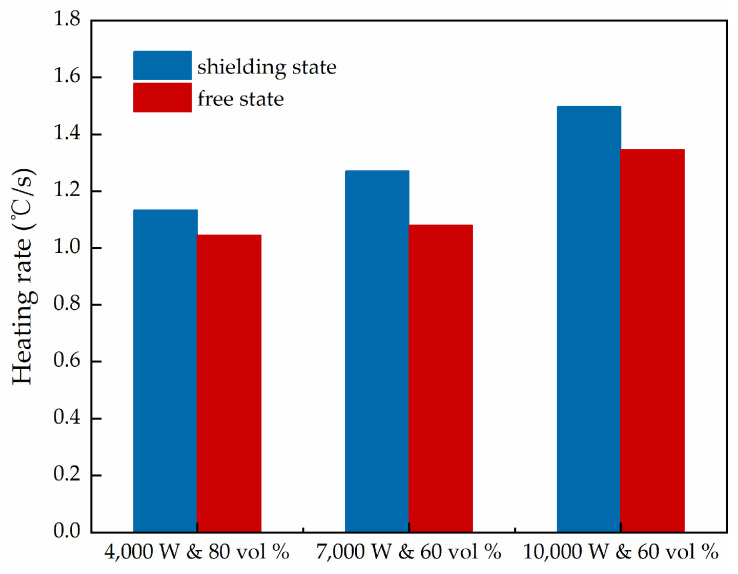
Surface heating rate (°C/s) for concrete slabs with different magnetite contents and different levels of power in the free state and with and bottom shielding.

The results of the experiment were collated and are shown in Figure 13 and Figure 14.

In Figure 13 and Figure 14, it can be seen that having a shield at the bottom of the microwave-absorbing cement concrete slab can enhance the rate of its increase in temperature through microwave absorption to some extent, and the microwave deicing efficiency was improved. The rate of the increase in temperature for 4000 W and 80 vol %, 7000 W and 60 vol %, and 10,000 W and 60 vol % with bottom shielding increased by 8.5 %, 17.6 % and 11.1 %, respectively, relative to the free unshielded state. The main reason for this is that, before entering the concrete, some of the microwaves are reflected into the air by the surface of the concrete slab, and the rest are absorbed by the microwave-absorbing sensitive material inside the concrete, leading to internal loss and conversion into heat, and then internal reflection and loss occur several times [37,38]. After reaching the bottom of the concrete slab, the microwaves that would have been transmitted out of the concrete are reflected by the shielding layer and continue to pass back to the inside of the concrete, and are again absorbed by the microwave absorbing material and converted into heat. That is, the concrete slab experiences two rounds of microwave heating, so its heating rate has a certain degree of improvement compared with that of the free state without shielding. The presence of a microwave shielding layer reduces the effects of microwave heating on the deeper structure of the road’s surface (basecourse layer, etc.), improving the safety of microwave radiation. The presence of the shielding layer improves the microwave heating efficiency of the microwave-absorbing concrete, which improves the deicing efficiency. In actual engineering, the efficiency of microwave deicing can be improved by mixing non-microwave-absorbing materials into the bottom layer as a shield.

In this section, the combination of 7000 W power and 60 vol % magnetite content was selected to test the effect of dry and wet conditions on the efficiency of microwave deicing compared with concrete specimens mixed with iron sulfide slag, steel slag, lead–zinc slag, and graphite to verify whether the microwave deicing effect of the combination of 7000 W and 60 vol % was significant under different environmental conditions.

### 3.5. Effect of Dry and Wet Conditions on the Efficiency of Microwave Deicing

**Figure 15:**
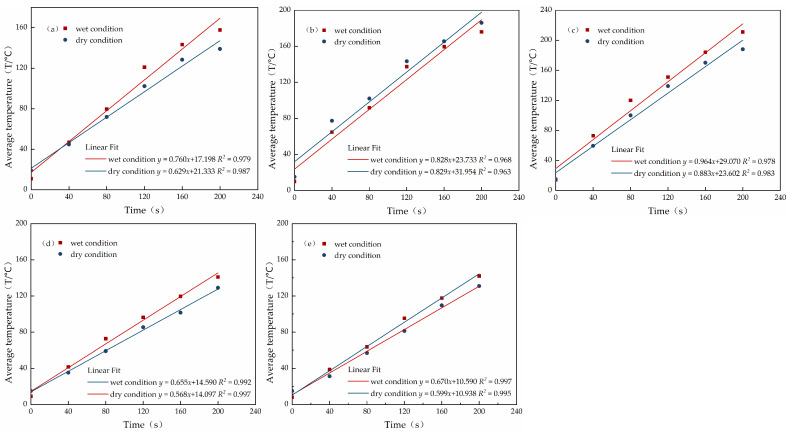
Fitted plots of average temperature of specimens of microwave-absorbing cement concrete made from different materials under dry and wet conditions: (**a**) magnetite; (**b**) iron sulfide slag; (**c**) steel slag; (**d**) lead–zinc slag; (**e**) graphite powder.

**Figure 16:**
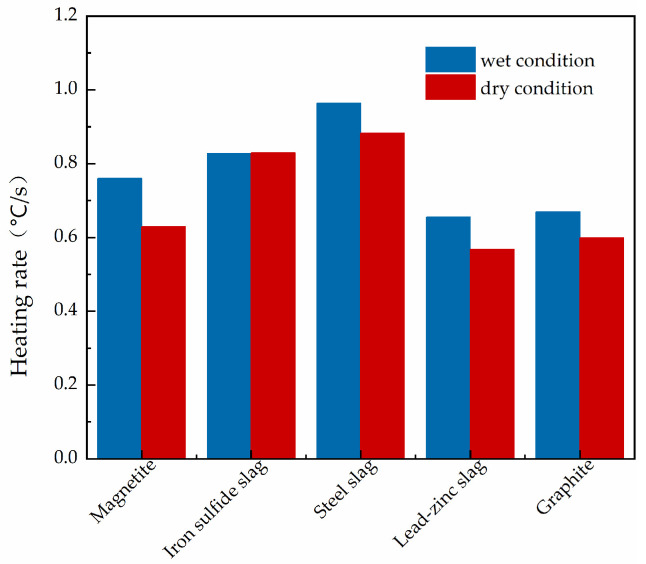
Heating rate of the surface of concrete slabs under dry and wet conditions for specimens of microwave-absorbing cement concrete made from different materials (°C/s).

The temperature versus time data of the five materials were collated, and the average temperature fitting plots were obtained after fitting, as shown in Figure 15. The heating rates of the five materials in the dry and wet states are shown in Figure 16.

In Figure 15 and Figure 16, it can be seen that the heating rates of magnetite concrete, steel slag concrete, lead–zinc slag concrete, and graphite concrete slab under wet conditions increased by 20.8%, 9.2%, 15.3%, and 11.9%, respectively, relative to those under dry conditions. The main reason is that water is a polar molecule and has a very strong ability to absorb microwaves. Therefore, when the absorbing microwave-concrete slab is in a wet state with a large water content, it can absorb a large number of microwaves and convert its loss to heat, so the absorbing concrete in a wet state will increase the efficiency of microwave-absorbing heat [39,40,41]. The heating rate of the wet iron sulfide slag concrete slab did not increase relative to that of the dry specimen, but was reduced. This is because the main component of iron sulfide slag is an iron oxide that easily reacts chemically with water, and then the content of the microwave absorbing component will be reduced, so the warming rate of the iron sulfide slag concrete slab was reduced in the wet state. Therefore, the microwave ice melting efficiency of microwave-absorbing concrete in the wet state was higher, and under 7000 W conditions, 60 vol % magnetite content of concrete compared to other microwave-absorbing materials in the wet state had better microwave absorption and warming performance.

## 4. Conclusions and Discussion

In recent years, the efficiency of microwave deicing has received wide attention from experts and scholars. In this study, we systematically examined the deicing laws of five microwave-absorbing materials, namely magnetite, iron sulfide slag, steel slag, lead–zinc slag, and graphite, under different influencing factors. Through the implementation of the pavement surface structure form experiments, microwave absorbing material content experiments, microwave power experiments, shielding state experiments, and dry and wet state experiments, we select the best combination to improve the deicing effect. The main conclusions are as follows.

(1) Concrete specimens with a scattered microwave-absorbing surface layer had more significant microwave absorption and warming advantages than the thin double-layered microwave-absorbing concrete specimens. A scattered microwave-absorbing layer with a thickness of 2 cm had the best deicing effect.
(2) The efficiency of microwave deicing increases significantly with an increase in the content of the microwave-absorbing material in the cement concrete, and the deicing time decreased with an increase in the content of the microwave-absorbing material, but there was also a decrease in the deicing rate. The deicing effect was good and economical when the content of magnetite was 40 vol %, 60 vol %, and 80 vol %.
(3) Increasing the microwave power can significantly improve the efficiency of microwave deicing. The difference Δt between the time when the ice starts to slide and the time when the ice has completely detached decreased significantly with an increase in the power of the incident microwaves.
(4) A shielding layer can be set at the bottom of the microwave-absorbing concrete slab to enhance its rate of heating by microwave absorption to a significant extent. Therefore, the structure of a microwave-absorbing concrete road surface can be designed with an absorbing layer above the reflective shielding layer, so the microwaves transmitted through the absorbing layer and to the shielding layer are reflected back to the upper absorbing layer, in order to achieve a second round of microwave heating.
(5) Microwave-absorbing concrete has a faster rate of warming in the wet state. Under 7000 W of power, a concrete slab with 60 vol % magnetite had a significant deicing effect compared with other materials.

In summary, this test concluded that 7000 W of power, a magnetite content of 60 vol %, and a scattered microwave-absorbing surface structure with a bottom shielding layer under wet conditions can greatly improve the efficiency of microwave deicing, and the microwave deicing effect is obvious.

This study provides a scientific basis for deicing technology for microwave-absorbing concrete road surfaces, which has a certain reference significance. In our study, we found that, although magnetite has a higher temperature uniformity compared to other microwave-absorbing materials, there are still temperature differences at different locations on the concrete slab surface during the experiment. Therefore, in the future, we will conduct research on the uniformity of warming of microwave-absorbing materials to maximize the deicing efficiency of microwave-absorbing concrete. In addition, we will further improve the experiments on the mechanical properties and durability of microwave-absorbing concrete indoors, to optimize the road performance of microwave-absorbing concrete.

## Figures and Tables

**Table 1:** Basic properties of the cement.

Density (g/cm^3^)	Specific Surface Area (m^2^/kg)	Coagulation Time (h)		Compressive Strength (MPa)		Flexural Strength (MPa)	
3.14	363	Initial condensation	Final condensation	3 d	28 d	3 d	28 d
		2 h 10 min	4 h 40 min	12.4	38.2	3.3	7.0

**Table 2:** Basic properties of the coarse aggregates of common gravel used in the test.

Apparent Density (g/cm^3^)	Crushing Value (%)	Needle Flake Content (%)	Water Absorption Rate (%)	Mud Content (%)	Stacking Density (g/cm^3^)
2.71	16.5	5.4	0.9	0.1	1.68

**Table 3:** Basic properties of the fine aggregate sand used in the test.

Density (g/cm^3^)	Stacking Density (g/cm^3^)	Modulus of Fineness	Mud Content (%)
2.61	1.52	2.78	1.1

**Table 4:** Basic properties of the microwave-absorbing materials (Table 4 shows the technical parameters of the raw materials, provided by the material purchase manufacturers, which are not part of the current study).

Material	Density (g/cm^3^)	Stacking Density (g/cm^3^)	Water Content (%)	Mohs Hardness	Melting Point (°C)	Heat Conductivity W/(m · k)	Abrasion Rate (%)
Magnetite	3.78	2.77	0	6	1594	0	0
Iron sulfide ore, 1–3 mm	3.762	0	0	>7	745	0.38	0
Iron sulfide ore, 5–10 mm	2.451						
Steel slag	3.62	1.785	0.54	0	1500	0	17.6
Lead–zinc slag	3.175	0	0.34	0	0	0.9	3.7
Graphite	2.26	0	0	0	3655	152	0

## Data Availability

Not applicable.
